# Prevalence and Influencing Factors of Severe Depression in Nurses During and After the COVID-19 Pandemic: A Large-Scale Multicenter Study

**DOI:** 10.1155/da/5727506

**Published:** 2024-12-20

**Authors:** Jiao Liu, Qinghe Liu, Lijie Ji, Yuan Yang, Ran Zhang, Yu Ding, Guoshuai Luo, Daliang Sun

**Affiliations:** Laboratory of Biological Psychiatry, Institute of Mental Health, Tianjin Anding Hospital, Mental Health Center of Tianjin Medical University, Tianjin 300222, China

**Keywords:** anxiety, COVID-19, nurse, severe depression, sleep disorders

## Abstract

**Background:** During the COVID-19 epidemic, nurses are facing tremendous psychological pressure. However, there is a lack of research on severe depression and its related factors in nurses after COVID-19. This study aimed to compare the prevalence and explore the influencing factors of severe depression among Chinese nurses during and after the COVID-19 pandemic.

**Methods:** This study recruited 937 frontline nurses during the outbreak and 784 frontline nurses who had participated in the initial recruitment after the pandemic. The self-rating depression scale (SDS), self-rating anxiety scale (SAS), and Pittsburgh Sleep Quality Index (PSQI) were used to assess subjects' depression, anxiety, and sleep disorders, respectively. Chi-square test, *t*-test, and binary logistic regression were used to identify factors influencing severe depression. Receiver operating characteristic (ROC) curves were used to assess the predictive value of severe depression-related variables.

**Results:** The study found that the incidence of severe depression among nurses after the epidemic (4.9%) was significantly higher than that during the epidemic (1.7%). In addition, academic qualifications, health status, frequency of exercise per week, anxiety, and sleep disorders were associated with severe depression among nurses after the COVID-19 epidemic. ROC analyses showed that SAS scores and PSQI scores had good predictive value for nurses' severe depression after the pandemic.

**Conclusions:** The prevalence of severe depression among nurses after COVID-19 is higher than that during COVID-19. Nurses continue to face severe psychological stress after the COVID-19 pandemic. Therefore, it is desperately needed for nurses to provide timely psychological support and establish a mental health support system after the COVID-19 epidemic.

## 1. Introduction

Nurses play a pivotal role amidst the COVID-19 pandemic, serving as frontline warriors in the medical field and facing immense pressure and risks [1–3]. The prolonged exposure to high-intensity and demanding work predisposes them to a higher incidence of mental health issues compared to the general population [3–5]. Consequently, the well-being of nurses during the COVID-19 epidemic is of utmost concern [6, 7]. Notably, studies have consistently reported a higher prevalence of depression among nurses compared to other healthcare workers during the pandemic outbreaks [8]. For example, ~31.1% of clinical nurses reported symptoms of depression during COVID-19 [9], a trend that persisted into subsequent stages [10].

Furthermore, the pre-existing global shortage of nurses, coupled with burnout experienced by nearly 50% of nurses before the pandemic, exacerbates the challenges faced during the COVID-19 crisis [11]. The intensified demands of caring for COVID-19 patients, alongside the complexities of managing routine patient care, amplify stress levels among nurses [6, 12]. Factors such as separation from family, sleep deprivation, policy changes, and increased workload contribute significantly to nurses' psychological distress during the epidemic [13]. Hence, understanding the incidence and determinants of severe depression among nurses is paramount for preserving their well-being, enhancing professional efficacy, and advancing healthcare delivery.

During the COVID-19 pandemic, healthcare workers, including nurses, were disproportionately affected by the psychological toll of the crisis. At the onset and peak of the COVID-19 pandemic, people around the world faced a sudden and unprecedented level of stress, accompanied by pronounced fear, anxiety, and a sense of unease [14]. The abrupt disruption to daily life, coupled with the threat of a highly contagious and largely unknown virus, created a pervasive atmosphere of uncertainty. For nurses, these challenges were amplified by their frontline roles. Over time, the prolonged nature of the pandemic led many to develop persistent psychological fatigue [15]. Even as the pandemic subsides, its psychological impact may linger in various forms, including feelings of loss, challenges in readjusting to social norms, anxiety about reintegration, and concerns over long-term health effects [16]. The phenomenon is not limited to the general population but is deeply felt among healthcare professionals.

Moreover, emerging evidence indicates that the psychological repercussions of the COVID-19 pandemic are enduring [10, 17–20]. Long COVID-19 refers to a range of symptoms that persist for weeks or even months after recovering from an acute COVID-19 infection [21]. A substantial proportion, ranging from 31% to 69%, of COVID-19 survivors experience persistent symptoms, imposing a significant load on their quality of life [22, 23]. The symptoms of long-term COVID-19 place a significant burden on the quality of life of COVID-19 survivors [24–27]. Long-term COVID-19 manifests with psychiatric symptoms, including fatigue, cognitive impairment, sleep disturbances, depression, anxiety, and posttraumatic stress disorder [28]. These symptoms significantly impact a patient's quality of life, physical or mental functioning, vitality, and overall resilience [29].

However, the focus on long-term COVID-19 continues to be lacking [30]. And, to our knowledge, no previous study has specifically compared severe depression and related factors among Chinese nurses during and after the COVID-19 pandemic. To bridge this gap, we conducted a multicenter survey involving frontline nurses during and after the COVID-19 pandemic. The study aimed to help guide strategies to address long-term COVID-19. The study's primary objectives were (1) to compare the prevalence of severe depression among Chinese nurses during and after the COVID-19 pandemic and (2) to explore the influencing factors affecting severe depression among nurses.

## 2. Methods

### 2.1. Study Design and Procedure

This large-scale multicenter study's sample consisted of nurses residing in China. The study designed followed the STROBE guidelines for observational studies.

Data on demographic characteristics, sleep disorders, depression, and anxiety were collected using the Questionnaire Star online survey platform. Informed consent was obtained from each participant prior to the survey. The study was conducted in accordance with the ethical standards set out in the 1964 Declaration of Helsinki and its subsequent amendments. This research was approved by the Institutional Review Board (IRB) of the Tianjin Mental Health Centre (approval number: 2023-027).

### 2.2. Participants

The study conducted its first large-scale online survey from November to December 2022 and repeated it with the same nurses from April to July 2023. Unlike other countries, China was still in the midst of a COVID-19 pandemic from November to December 2022. As China begins to liberalize its closure policy, April to July 2023 is after the COVID-19 pandemic.

**Figure 1:**
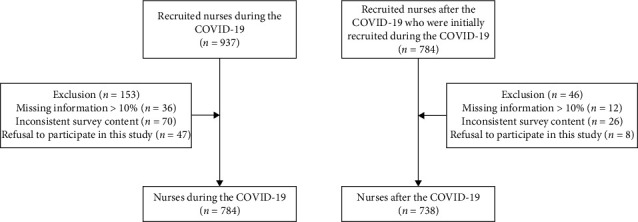
Flowchart of this study.

Inclusion criteria: (1) nurses working on the front line during and after the COVID-19 pandemic and after and (2) understanding of the purpose of the survey, informed consent, and voluntary participation in the study. Exclusion criteria: (1) inconsistent responses to survey content, (2) missing data of more than 10%, and (3) refusal to participate in the study. The flowchart of this study is shown in Figure 1.

### 2.3. Assessment Measures

A self-designed general demographic questionnaire was used, which consisted of questions that included nurses' general demographic characteristics, work characteristics, and perceptions of the impact of the COVID-19 pandemic on their lives and work. For example, the number of exercise sessions per week was defined as more than 30 min per session, which was defined as 1 session; divided into 0, 1–2, and ≥3 times per week. In addition, we investigated whether nurses perceived COVID-19 as disturbing their lives and whether they were concerned that their clinical work could cause COVID-19 infections.

The Pittsburgh Sleep Quality Index (PSQI) was employed to evaluate sleep disorders [31]. This comprehensive assessment tool comprises seven domains: subjective sleep quality, sleep latency, sleep duration, habitual sleep efficiency, sleep disturbances, use of sleep medications, and daytime dysfunction. Each domain is scored on a scale of 0–3, with a total PSQI score ranging from 0 to 21. A total score of ≥8 indicates the presence of a sleep disorder [32]. Anxiety levels were assessed using the self-rating anxiety scale (SAS) [33], while depression symptoms were evaluated using the self-rating depression scale (SDS) [34]. Severe depression was defined by a standardized SDS score equal to or greater than 73 [35].

### 2.4. Statistical Analyses

Statistical analyses were performed using SPSS 23.0. Categorical variables were expressed as frequencies and percentages, and continuous variables were expressed as means (M) and standard deviations (SD). Comparisons between categorical and continuous variables during and after the COVID-19 epidemic were performed using the chi-square test and *t*-test, respectively. Significance was determined at *p* < 0.05. Binary logistic regression analyses were used to assess the effect of different factors on severe depression, with the presence of severe depression as the dependent variable and factors showing significant differences in the chi-square test or *t*-test as independent variables. Results were expressed as odds ratio (OR) and 95% confidence interval (95% CI). Receiver operating characteristic (ROC) curve analyses were performed using GraphPad Prism 9.0 to assess the predictive value of the variables of interest for severe depression.

## 3. Results

### 3.1. Comparison of Demographic Characteristics of Participants

In this study, 784 nurses were recruited during the epidemic, and 738 nurses were recruited after the epidemic.  Table 1 compares the differences between the participants' nurses during and after the epidemic. More nurses (4.9%) were severely depressed after the epidemic than under the epidemic (1.7%). In addition, after COVID-19, health status, COVID-19 virus infection status, and disruption of life were significantly higher than during the outbreak (*p* < 0.01). However, the proportion of permanent staff nurses and the frequency of exercise per week significantly decreased after the COVID-19 epidemic (*p* < 0.01).

### 3.2. Comparison of SAS, SDS, and PSQI Scores of Nurses During and After the COVID-19 Epidemic

Table 2 shows the differences in SAS, SDS, and PSQI scores during and after the epidemic. The mean total scores of the SAS, SDS, and PSQI were significantly lower after the epidemic compared to during the epidemic (*p* < 0.001). In addition, PSQI total scores, sleep duration, sleep disorder, and sleep medications were significantly lower for after-COVID-19 epidemic nurses than that during the epidemic (*p* < 0.01). However, subjective sleep quality scores were significantly higher after the epidemic than during the epidemic (*p* < 0.01). After Bonferroni correction, there were still significant differences in SAS scores, SDS scores, total PSQI scores, sleep duration, sleep disorder, and sleep medications (*p* Bonferroni < 0.05/10 = 0.005).

### 3.3. Factors Influencing Severe Depression in Nurses

As shown in Table 3, binary logistic regression analyses were performed by categorizing the covariates for severe depression (SDS ≥ 73). The results showed that during the COVID-19 epidemic, there were no significant effects on the emergence of severe depression among nurses. However, after the COVID-19 epidemic, nurses in ordinary health were 10.555 times more likely to be at risk of severe depression than those in the well (OR = 10.555, 95% CI 2.124–52.449). Interestingly, nurses with a bachelor's degree had 3.951 times the risk of severe depression than those with associate college and below (OR = 3.951, 95% CI 1.067–14.629). In addition, nurses with an exercise frequency of 1–2 times per week had 0.253 times the risk of severe depression than those who did not exercise per week (OR = 0.253, 95% CI 0.084–0.764).

**Figure 2:**
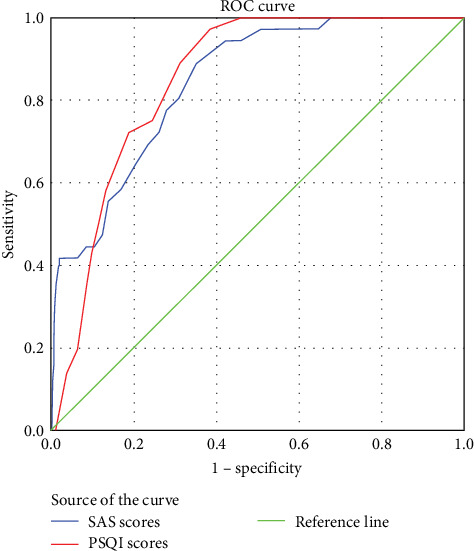
ROC analysis of the factors influencing nurses' severe depression after the COVID-19 pandemic. The area beneath the curve was 0.844 for SAS scores and 0.851 for PSQI scores. PSQI, Pittsburgh Sleep Quality Index; ROC, receiver operating characteristic curve; SAS, self-rating anxiety scale.

**Figure 3:**
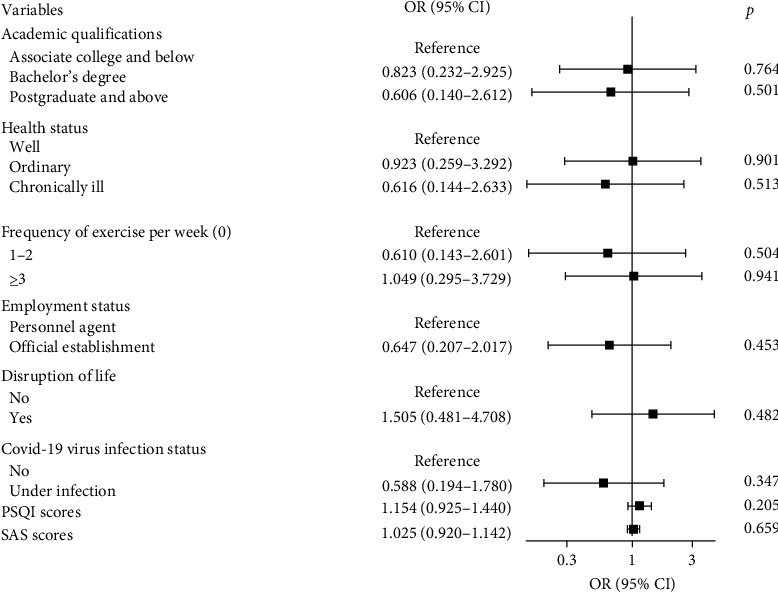
Forest plot of factors associated with severe depression in nurses during the COVID-19 epidemic. CI, confidence interval; OR, odds ratio; PSQI, Pittsburgh Sleep Quality Index; SAS, self-rating anxiety scale.

**Figure 4:**
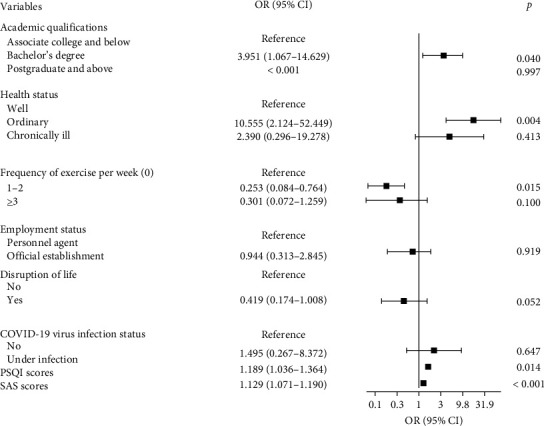
Forest plot of factors associated with severe depression in nurses after the COVID-19 epidemic. CI, confidence interval; OR, odds ratio; PSQI, Pittsburgh Sleep Quality Index; SAS, self-rating anxiety scale.

Furthermore, anxiety (OR = 1.129, 95% CI 1.071–1.190) and sleep disorders (OR = 1.189, 95% CI 1.036–1.364) emerged as independent risk factors for severe depression among nurses after the pandemic. ROC analyses showed that SAS scores and PSQI scores exhibited good predictive value for nurses' severe depression after the pandemic (the area under the ROC curve was 0.844 for SAS scores and 0.851 for PSQI scores, respectively) (Figure 2). Additionally, Figure 3 presents a forest plot of factors associated with severe depression in nurses during the COVID-19 epidemic, while Figure 4 displays a forest plot of factors associated with severe depression in nurses after the epidemic.

## 4. Discussion

We found that the prevalence of severe depression among nurses was significantly higher after the COVID-19 pandemic than during the pandemic. This result suggests that nurses continue to face severe psychological stress after the pandemic, highlighting that this effect persists long after the epidemic has finished [36–38]. This result manifests as long-COVID-19 or post-COVID-19 syndrome [38–40]. The mechanisms underlying the relationship between long-term COVID-19 and increased stress are still being explored, but several studies have proposed potential explanations. For instance, long-term COVID-19 may lead to neuroinflammation, which can disrupt brain function, particularly in areas involved in emotion regulation and stress response [41]. Additionally, research has shown that many individuals recovering from COVID-19 report reduced heart rate variability (HRV), a condition often linked to heightened stress, anxiety, and depression [42]. Furthermore, long-term COVID-19 patients tend to have elevated levels of inflammatory markers in their blood, which are associated with the development of psychiatric symptoms, including anxiety and depression [43]. Moreover, these patients may experience social isolation and a decline in quality of life, which can contribute to increased psychological distress and anxiety [44].

Interestingly, however, while the number of people with severe depression increased after the pandemic, the average SDS score actually decreased after the pandemic. This phenomenon may be related to changes in the sample distribution. Some individuals experienced intense psychological shock at the onset of the pandemic [14, 45]. However, over time, many gradually recovered or strengthened their psychological resilience through adaptation or by relying on social support systems [46, 47]. As a result, the proportion of individuals with mild or moderate depression may have decreased, contributing to a lower overall SDS mean score. Additionally, patients with severe depression carry a heavier psychological burden, and the pandemic has had a more pronounced impact on their quality of life [48]. This may have made them more prominent in postpandemic studies [49]. Furthermore, the heightened social attention during the pandemic may have led individuals to emphasize severe symptoms while overlooking or underreporting mild ones. In fact, the observed increase in severe depression following the pandemic does not necessarily indicate a significant decline in the overall mental health of the population. Actually, it may reflect a worsening trend within specific subgroups.

During the COVID-19 pandemic, there was no significant relationship between nurses' academic qualifications and severe depression. However, postpandemic, higher academic qualifications were associated with higher levels of severe depression. There is a strong relationship between academic standards and mental health [50, 51]. Higher academic qualifications mean more years of education, older age, and less work experience, which can cause an increased risk of depression levels [52]. Higher academic qualifications usually require more study and competition and face more social pressure, which can lead to mental health problems such as depression [53]. It is essential to prioritize the mental health and professional growth of highly educated nurses in the postpandemic period.

Additionally, we found increased levels of severe depression among nurses with poorer health after the COVID-19 pandemic. One study showed that for each additional chronic condition, the odds of developing depression were 45% higher [54]. When people get sick, they are more likely to feel tired, frustrated, and helpless, which can exacerbate depressive moods [55]. Our study found that nurses who exercised weekly had lower levels of severe depression than nurses who did not have an exercise routine. Exercise helps to improve cardiovascular health, boost the immune system, promote sleep quality, and control weight, all of which have been linked to depression relief [56, 57]. Exercise has been linked to depression relief by promoting overall physical and mental well-being.

In addition to physical activity, mental health conditions like anxiety and sleep disorders are also closely associated with severe depression. Our study also found that anxiety and sleep disorders in nurses after the COVID-19 pandemic were associated with severe depression [58, 59]. Anxiety and sleep disorders are strongly associated with severe depression [60]. Anxiety and depression are often comorbid, and people with depression usually have coexisting sleep disorders [61]. Anxiety and sleep disorders may exacerbate depressive symptoms, which in turn may lead to anxiety and sleep problems [62]. This suggests that nurses' mental health problems are often interrelated and may require comprehensive mental health interventions to cope.

In developing intervention strategies targeting nurses' mental health, we suggest focusing on the following aspects: first, strengthening mental health education to enhance nurses' awareness of mental health problems and their ability to cope with them, and helping nurses to establish a healthy work–life balance [63]; second, hospitals should ensure that nurses have timely access to health support and provide relevant resources and information, including regular medical checkups and health education [64]; third, nurses should be encouraged to adopt a healthy lifestyle and actively participate in sports and other healthy activities [65]; finally, management should establish a comprehensive mental health support system to provide nurses with timely mental health services and support, including psychological counseling, psychotherapy or psychoeducation [66].

This study offers a preliminary look at severe depression among Chinese nurses during and after the COVID-19 pandemic, but there are still some limitations. First, the study relied on self-reported data, which may have introduced bias, and the findings might not have been generalizable to all nurse populations beyond the surveyed regions. Second, as with any observational study, causality could not be inferred, and the possibility of unmeasured confounding variables influencing the results could not be ruled out. Finally, the study assessed only nurses in China, making it challenging to generalize the results to the entire nurse population.

In summary, we found that the prevalence of severe depression is higher post-COVID-19 pandemic compared to during the pandemic. Anxiety and sleep disorders are risk factors for severe depression among nurses after the COVID-19 pandemic. Future studies can further explore the mechanisms of long-term COVID-19 effects on mental health and strengthen interdisciplinary research and cooperation.

## Figures and Tables

**Table 1:** Comparison of nurses' severe depression during and after the COVID-19 pandemic.

Variables	During the COVID-19 epidemic	After the COVID-19 epidemic	*X* ^2^	*p*
Gender				
Male	395 (50.4%)	366 (49.6%)	0.095	0.758
Female	389 (49.6%)	372 (50.4%)		
Employment status				
Personnel agent	403 (51.4%)	521 (70.6%)	58.709	**<0.001**
Official establishment	381 (48.6%)	217 (29.4%)		
Academic qualifications				
Associate college and below	263 (33.5%)	254 (34.4%)	130.608	**<0.001**
Bachelor's degree	286 (36.5%)	403 (54.6%)		
Postgraduate and above	235 (30.0%)	81 (10.9%)		
Personality traits				
Extraversion	371 (47.3%)	325 (44.0%)	1.651	0.199
Introversion	413 (52.7%)	413 (56.0%)		
Health status				
Well	264 (33.7%)	325 (44.0%)	80.510	**<0.001**
Ordinary	262 (33.4%)	314 (42.5%)		
Chronically ill	258 (32.9%)	99 (13.4%)		
Frequency of exercise per week				
0	268 (34.2%)	309 (41.9%)	39.215	**<0.001**
1–2	254 (32.4%)	287 (38.9%)		
≥3	262 (33.4%)	142 (19.2%)		
COVID-19 virus infection status				
No	266 (33.9%)	83 (11.2%)	110.669	**<0.001**
Under infection	518 (66.1%)	655 (88.8%)		
Disruption of life				
No	373 (47.6%)	159 (21.5%)	113.305	**<0.001**
Yes	411 (52.4%)	579 (78.5%)		
Worried about potential infection				
No	398 (50.8%)	398 (53.9%)	1.526	0.217
Yes	386 (49.2%)	340 (46.1%)		
Severe depression or not				
No	771 (98.3%)	702 (95.1%)	12.649	**<0.001**
Yes	13 (1.7%)	36 (4.9%)		

*Note:* The question of “Disruption of life”: Has your life been disrupted by COVID-19? Bolding indicates *p* < 0.05.

**Table 2:** Comparison of SAS, SDS, and PSQI scores of nurses during and after the COVID-19 pandemic (mean ± SD).

Variables	During the COVID-19 epidemic	After the COVID-19 epidemic	*t*	*p*
SAS scores	69.61 ± 5.04	51.54 ± 12.15	37.472	**<0.001**
SDS scores	62.48 ± 4.69	55.63 ± 12.57	13.913	**<0.001**
PSQI total scores	10.68 ± 2.53	9.37 ± 4.67	6.739	**<0.001**
Subjective sleep quality	1.36 ± 1.01	1.50 ± 1.08	−2.740	**0.006**
Sleep latency	1.63 ± 0.76	1.68 ± 0.93	−1.151	0.250
Sleep duration	1.56 ± 1.01	1.28 ± 0.85	5.863	**<0.001**
Habitual sleep efficiency	1.20 ± 0.75	1.17 ± 0.76	0.770	0.442
Sleep disorder	1.89 ± 0.37	1.53 ± 0.76	11.526	**<0.001**
Sleep medications	1.45 ± 1.00	0.60 ± 0.89	17.526	**<0.001**
Daytime dysfunction	1.59 ± 0.73	1.60 ± 1.04	−0.240	0.810

*Note:* Bolding indicates *p* < 0.05.

Abbreviations: PSQI, Pittsburgh Sleep Quality Index; SAS, self-rating anxiety scale; SD, standard deviations; SDS, self-rating depression scale.

**Table 3:** Logistic regression analysis of the influencing factors of nurses' severe depression.

Influence factors	During the COVID-19 epidemic			After the COVID-19 epidemic		
	*B*	*p*	OR (95% CI)	*B*	*p*	OR (95% CI)
Constant	−6.609	0.115	0.001	−14.365	**<0.001**	**<0.001**
Academic qualifications (associate college and below)			**Ref.**			**Ref.**
Bachelor's degree	−0.194	0.764	0.823 (0.232–2.925)	1.374	**0.040**	3.951 (1.067–14.629)
Postgraduate and above	−0.501	0.501	0.606 (0.140–2.612)	−16.997	0.997	<0.001
Health status (well)			**Ref.**			**Ref.**
Ordinary	−0.081	0.901	0.923 (0.259–3.292)	2.357	**0.004**	10.555 (2.124–52.449)
Chronically ill	−0.484	0.513	0.616 (0.144–2.633)	0.871	0.413	2.390 (0.296–19.278)
Frequency of exercise per week (0)			**Ref.**			**Ref.**
1–2	−0.494	0.504	0.610 (0.143–2.601)	−1.374	**0.015**	0.253 (0.084–0.764)
≥3	0.048	0.941	1.049 (0.295–3.729)	−1.199	0.100	0.301 (0.072–1.259)
Employment status (personnel agent)	−0.436	0.453	0.647 (0.207–2.017)	−0.057	0.919	0.944 (0.313–2.845)
Disruption of life (no)	0.409	0.482	1.505 (0.481–4.708)	−0.871	0.052	0.419 (0.174–1.008)
COVID-19 virus infection status (no infection)	−0.531	0.347	0.588 (0.194–1.780)	0.402	0.647	1.495 (0.267–8.372)
PSQI scores	0.143	0.205	1.154 (0.925–1.440)	0.173	**0.014**	1.189 (1.036–1.364)
SAS scores	0.024	0.659	1.025 (0.920–1.142)	0.121	**<0.001**	1.129 (1.071–1.190)

*Note:* Bolding indicates *p* < 0.05. The question of “Disruption of life”: Has your life been disrupted by COVID-19?

Abbreviations: CI, confidence interval; OR, odds ratio; PSQI, Pittsburgh Sleep Quality Index; Ref., reference; SAS, self-rating anxiety scale.

## Data Availability

The data that support the findings of this study are available from the corresponding author upon reasonable request.
